# Overweight and obesity and its socio-demographic correlates among urban Ethiopian women: evidence from the 2011 EDHS

**DOI:** 10.1186/s12889-016-3315-3

**Published:** 2016-07-26

**Authors:** Solomon Abrha, Solomon Shiferaw, Kedir Y. Ahmed

**Affiliations:** 1 Department of Epidemiology and Biostatistics, School of Public Health, College of Medicine and Health Science, Wolayta Sodo University, PO Box: 138, Wolayta Sodo, Ethiopia; 2 School of Public Health, College of Health Sciences, Addis Ababa University, PO Box: 9086, Addis Ababa, Ethiopia; 3 Department of Public Health, College of Medicine and Health Science, Debre Markos University, PO Box: 269, Debre Markos, Ethiopia

**Keywords:** Overweight, Obesity, Urban Ethiopian women

## Abstract

**Background:**

Evidences show that the burden of overweight and obesity is increasing in developing countries, particularly among urban women. Despite this worrying trend and the recognition of the emerging problem of chronic diseases in the recently launched Health Sector Transformation Plan of Ethiopia, little efforts are being made to address overweight and obesity. The present study aimed at assessing the prevalence and socio-demographic correlates of overweight and obesity among urban women.

**Methods:**

This study was based on the 2011 Ethiopian Demographic Health Survey (EDHS) that used a two-stage stratified cluster sampling technique. A total of 3602 non-pregnant urban reproductive age women were included in the analysis. Simple descriptive, bivariate and multiple logistic regression analysis were employed as appropriate.

**Results:**

The prevalence of overweight and obesity among urban Ethiopian women was found to be 435 (12.1 %) and 99 (2.8 %), respectively. Urban women in the age groups from 20–29 years [Adjusted Odds Ratio (AOR) = 2.3 95 % CI: 1.4, 3.9], 30–39 years (AOR = 5.0 95 % CI: 2.9, 8.8) and 40–49 years (AOR = 9.8 95 % CI: 5.1, 13.8) were significantly more likely to have overweight and obesity compared to the youngest age group (15 to 19 years). The odds of being overweight and obese was significantly higher among women in the richest quintile (AOR = 1.8 95 % CI: 1.1, 2.5), those with secondary and above education (AOR = 2.0 95 % CI: (1.3, 3.1) and married women (AOR = 2.0 95 % CI: (1.2, 3.3).

**Conclusions:**

The prevalence of overweight and obesity was found to be higher in urban women compared to the national average. Being married, older, belonging to the richest quintile, living in the three metropolises (Addis Ababa, Harari and Dire Dawa), and with secondary and above educational level are independent predictors of overweight and obesity. Programs that target on older, educated and well to do women, and those living in the big cities are expected to cope with this substantial public health concern.

## Background

The global prevalence of overweight and obesity has been significantly increasing over the past four decades. The combined prevalence of overweight and obesity has increased by 27.5 % for adults and 47.1 % for children between 1980 and 2013. During this period, the proportion of women with overweight and obesity has also increased from 29.8 to 38.0 % [1]. Excess body weight is a major risk factor for mortality and morbidity from cardiovascular diseases, diabetes, cancers and musculo-skeletal disorders [2, 3]. In 2010, it was estimated that 3.4 million global annual deaths, 3.9 % of years of life lost and 3.8 % of global disability adjusted life years (DALYs) were caused by overweight and obesity [1, 4, 5]. Once associated only with high-income countries, overweight and obesity are now also prevalent in low and middle-income countries and more noticeably in urban areas [6–8].

Evidences show that the burden of overweight and obesity is increasing in developing countries, particularly among urban women. Also, women in developing countries have higher rates of overweight and obesity than men and this relationship persisted over time [1, 9–11]. Women’s overweight doubled from 9 % in 1980 to 18 % in 2008 while obesity more than doubled from 2 to 5 % over the same time period [12]. The rates are also increasing at alarming rates in Sub-Saharan Africa [13]. In 2013, the prevalence of overweight and obesity among women in Eastern Sub-Saharan African countries was 23.7 and 8.8 %, respectively [1]. Similarly, a Nigerian study based on demographic and health survey data showed, about 36.4 % of urban women had either overweight or obesity [14].

A number of studies conducted on chronic diseases risk factors showed an increase in the prevalence of overweight and obesity in Ethiopia [15–19]. A study based on Ethiopian Demographic Health Survey (EDHS) data showed that the prevalence of overweight increased by 28 % (from 16.1 % in 2000 to 20.6 % in 2011) among women in Addis Ababa [20]. A similar study showed that one-third of female permanent commercial bank employees and public school teachers in Addis Ababa were either overweight or obese [19].

Despite this worrying trend and the recognition of the emerging problem of chronic diseases as a national public health concerns in the recently launched Health Sector Transformation Plan of Ethiopia [21], little efforts are being made to address overweight and obesity [3]. Moreover, given the fact that the changes favoring unhealthy dietary pattern and limited physical activity are associated with urbanization, the prevalence of overweight and obesity in urban areas is consistently higher compared to rural areas [22–24]. Therefore, the present study aimed at assessing the prevalence and socio-demographic correlates of overweight and obesity among urban non-pregnant reproductive age women in Ethiopia.

## Methods

### Study design and data sources

This cross-sectional study was based on the 2011 Ethiopia Demographic and Health Survey (EDHS), which was designed as a nationally representative survey. The survey provided data on a wide range of indicators relating to population, health, and nutrition. It was funded by the United States Agency for International Development, the Government of Ethiopia with other stake holders and implemented out by the Central Statistical Agency (CSA).

### Sampling techniques and population

The survey employed two-stage stratified cluster sampling technique. The country is structured into nine regional states and two City Administrations Councils (Addis Ababa and Dire Dawa). Stratification was achieved by separating each region and one of the town administrations into urban and rural areas, except the entirely urban Addis Ababa. In total, 23 sampling strata have been created. Each stratum was further divided into enumeration areas (EAs) using the list of all EAs (clusters) prepared by the 2007 Population and Housing Census (PHC) as a sampling frame. In the first stage, a total of 624 EAs were selected, of which 187 were from urban areas. The EAs were selected with probability proportional to the EA size and with independent selection in each sampling stratum. Households comprised the second stage of sampling in which a fixed number of 30 households were selected for each EAs [25, 26]. Women who were pregnant and those with outlier BMI value < 12.00 kg/m^2^ and > 50.00 kg/m^2^, were excluded from this study. Overall, 3, 602 urban non-pregnant women of aged 15–49 years were included in the final analysis.

### Data collection

The data collection procedure for the 2011 Ethiopian Demographic and Health Survey was documented in the full report [26]. Before the actual analysis was carried out the dataset was explored by running frequencies of available predictor variables, and weight/height variables. During exploration, it was noted that weight or height were measured for 3606 urban non-pregnant women. Of these, two outlier cases and two flagged cases were excluded from the final analysis. Socio-demographic characteristics of the women such as region of residence, age, marital status, educational status, occupation, wealth status and parity were the predictor variables for which data were collected.

### Operational definitions

- Body mass index (BMI): Weight in kilograms divided by height in meters squared.
- Overweight: BMI > = 25.00 Kg/m^2^ and < = 29.99 Kg/m^2^
- Obese: BMI > = 30.00 Kg/m^2^ and < = 50.00 kg/m^2^
- Overweight and obese: BMI > = 25.00 Kg/m^2^ and < = 50.00 Kg/m^2.^ The outcome variable was dichotomized as: Overweight and obese (Yes) compared to the reference of normal and underweight groups (No).
- Region of residence was recoded as ‘The three Metropolis’ (which include Addis Ababa, Harari and Dire Dawa), Tigray, Amhara, Oromia, SNNPR and “Other regions” (which include Afar, Benshangul-Gumuz, Gambela and Somali).
- Kebele: the smallest administrative unit in Ethiopia.
- Woreda (district): an administrative unit which consists of kebeles
- EA (Clusters): An EA is a geographic area consisting of a convenient number of dwelling units which served a counting unit for the census.
- Flagged cases: inconsistent measurements noted for weight and height.

### Statistical analysis

Complex sample analysis was performed using SPSS version 16.0 statistical software to account for the multi-stage cluster study design. The design variables of primary sampling unit (EAs) as cluster and stratification variable (residence) as strata were used. Analysis was carried out based on the weighted count. Sample weight was an eight digit variable with six implied decimal places and was divided by 1,000,000 before applying the weighting factor.

Frequencies and proportions were computed for description of the study population in relation to selected socio-demographic variables. Wealth index quintiles were constructed using principal component analysis. Multiple logistic regression analysis was used to identify independent predictors and control for confounding. Results were presented in the form of odds ratios (OR) and 95 % confidence intervals. Statistical significance was set at a p-value of 0.05. The overall model fitness test statistics with adjusted Wald chi-square value of 74.263 (*p* value < 0.0001) shows the fitted model performance was better than the null model.

## Results

### Characteristics of respondents

In this study, the proportion of urban women aged 20–29 years was 1438 (39.9 %) with a mean (± SD) age of 26.4 (± 0.2) years. About 893 (24.8 %), 857 (23.8 %), 477 (13.2 %) and 280 (7.8 %) of them were living in Amhara, Oromia, SNNPR, and Tigray regions, respectively. Among participants, 1542 (42.8 %) had only primary level education, and 1602 (44.5 %) married while 798 (22.1 %) were in the poorest quintile. Nearly half (47.9 %) had no child (Table 1).

**Table 1:** Characteristics of non-pregnant reproductive age women in urban Ethiopia, 2011 EDHS, (N^w^ = 3602)

Variables	Frequency (%)
Age Group^§^	
15–19	996 (27.7)
20–29	1438 (39.9)
30–39	783 (21.7)
40–49	385 (10.7)
Region of residence	
Tigray	280 (7.8)
Three Metropolis	886 (24.6)
Amhara	893 (24.8)
Oromia	857 (23.8)
SNNPR*	477 (13.2)
Other Regions	209 (5.8)
Wealth quintiles	
Poorest	798 (22.1)
Poorer	674 (18.7)
Middle	712 (19.8)
Richer	709 (19.7)
Richest	709 (19.7)
Educational status	
No formal education	781 (21.7)
Primary (grade 1–8^th^)	1542 (42.8)
Secondary and above (grade 9^th^+)	1279 (35.5)
Parity	
No child	1726 (47.9)
1–4 children	1485 (41.2)
5+ children	391 (10.9)
Marital status	
Single	1523 (42.3)
Married	1602 (44.5)
Divorced/widowed	477 (13.2)
Working status	
Not working	1509 (41.9)
White-collar Work	1450 (40.3)
Manual Work	619 (17.1)
Others	24 (0.7)
Nutritional status	
Underweight	718 (19.9)
Normal	2350 (65.2)
Overweight	435 (12.1)
Obesity	99 (2.8)

*SNNPR = Southern Nations and Nationalities and Peoples Region

N^W^ = Total weighted count excluding pregnant women and women with extreme BMI values (<12.00 kg /m2 and > 50.00 kg /m2)

§ = age classification used in EDHS reports (2005 and 2011) under maternal nutritional status section

### Prevalence of overweight and obesity and its socio-demographic correlates

The prevalence of overweight and obesity was found to be 435 (12.1 % 95 % CI: 10.1, 14.4) and 99 (2.8 % 95 % CI: 2.1, 3.6), respectively. Thus, about 534 (14.9 %, 95 % CI: 12.4, 17.6) of urban reproductive age women were overweight and obese (Table 1).

From the multivariable logistic regression analysis; age, region of residence, wealth index, educational status and marital status were significantly associated with being overweight and obese.

The likelihood of being overweight and obese was higher among 20–29 years (AOR = 2.3 95 % CI: 1.4, 3.9), 30–39 years (AOR = 5.0 95 % CI: 2.9, 8.8) and 40–49 years (AOR = 9.8 95 % CI: 5.1, 13.8) of age groups compared to the reference age group of 15–19 year olds. With regards to regional distribution, the odds of being overweight and obese was higher among residents of the three Metropolis (AOR = 2.2 95 % CI: 1.2, 4.2) and among residents of Southern Nations and Nationalities and Peoples Region (SNNPR) (AOR = 2.6 95 % CI: 1.2, 5.8) compared to urban women in Tigray (Table 2).

**Table 2:** Socio-demographic correlates of overweight and obesity among urban reproductive age women in Ethiopia, 2011 EDHS (N^W^=3602)

Variables	Overweight and obesity		Crude Odds Ratio (95 % CI)	Adjusted Odds Ratio (95 % CI)
	Yes (n, %)	No (n, %)		
Age Group^§^				
15–19	44 (4.4)	952 (95.6)	1	1
20–29	183 (12.7)	1255 (87.3)	3.2 (2.0, 5.0)*	2.3 (1.4, 3.9)*
30–39	183 (23.4)	600 (76.6)	6.7 (4.3, 10.5)*	5.0 (2.9, 8.8)*
40–49	124 (32.2)	261 (67.8)	10.3 (8.4, 16.6)*	9.8 (5.1, 13.8)*
Region of Residence				
Tigray	24 (8.6)	256 (91.4)	1	1
Three Metropolis	180 (20.3)	706 (79.7)	2.7 (1.4, 5.1)*	2.2 (1.2, 4.2)*
Amhara	72 (8.1)	821 (91.9)	0.9 (0.4, 2.3).	0.9 (0.3, 2.4)
Oromia	121 (14.1)	736 (85.9)	1.7 (0.8, 3.9)	1.5 (0.6, 3.2)
SNNPR^*^	97 (20.3)	380 (79.7)	2.7 (1.2, 6.2)*	2.6 (1.2,5.8)*
Others	40 (19.1)	169 (80.9)	2.5 (1.2, 5.3)*	2.5 (1.1, 5.4)*
Wealth Quintile				
Poorest	84(10.5)	714 (89.5)	1	1
Poorer	105 (15.6)	569 (84.4)	1.6 (1.0, 2.4)	1.1 (0.7,1.8)
Middle	140 (19.7)	572 (80.3)	2.1 (1.2, 3.8)*	1.6 (0.9,2.9)
Richer	66 (9.3)	643 (80.4)	0.9 (0,5, 1.6)	0.9 (0.5, 1.6)
Richest	139 (19.6)	570 (83.9)	2.1 (1.3, 3.3)*	1.8 (1.1, 2.5)*
Educational Status				
No formal education	103 (13.2)	678 (86.8)	1	1
Primary (1–8^th^)	202 (13.1)	1340 (86.9)	1.0 (0.7, 1.4)	1.5 (1.0, 2.3)
Secondary+	229 (17.9)	1050 (82.1)	1.4(0.9, 2.2)	2.0 (1.3, 3.1)*
Parity				
No child	143 (8.3)	1583 (91.7)	1	1
1–4 children	312 (21.0)	1173 (79.0)	3.0 (2.1, 4.1)*	1.1 (0.7, 1.7)
5+ children	79 (20.2)	312 (79.8)	2.8 (1.9, 4.2)*	0.7 (0.4,1.3)
Marital Status				
Single	111 (7.3)	1412 (92.7)	1	1
Married	351 (21.9)	1251 (78.1)	3.6 (2.5, 5.1)*	2.0 (1.2, 3.3)*
Divorced/widowed	72 (15.1)	405 (84.9)	2.3 (1.5,3.5)*	1.2 (0.6, 2.3)
Occupational status				
Not working	206 (13.7)	1303 (86.3)	1	1
White- collar work	258 (17.8)	1192 (82.2)	1.4 (0.9,1.9)	1.0 (0.7, 1.4)
Manual Work	68 (11.0)	551 (89.0)	0.8 (0.5, 1.3)	0.8 (0.5, 1.2)

*SNNPR = Southern Nations and Nationalities and Peoples Region

N^W^ = Total weighted count excluding pregnant women and women with extreme BMI values (<12.00 kg /m^2^ and > 50.00 kg /m^2^)

§ = age classification used in EDHS reports (2005 and 2011) under maternal nutritional status section

The odds of overweight and obesity was 1.8 times (95 % CI: 1.1, 2.5) higher among those who are in the richest quintile compared to those who are in the poorest quintile. Having a Secondary and above educational level (AOR = 2.0 95 % CI: (1.3, 3.1) and being married (AOR = 2.0 95 % CI: (1.2, 3.3) were significantly and positively associated with the condition of being overweight and obese (Table 2).

## Discussion

The findings of this study showed that the overall prevalence of overweight and obesity among urban Ethiopian women was 14.9 %, which was considerably higher compared to the national average of 5.7 % [26]. However, it is lower than other pocket study conducted in Southwest part of Ethiopia (23.4 %) [18]. Previous studies from the capital, Addis Ababa and among workers of Wonji Shewa sugar factory reported a much higher figure that ranged from 26.7 to 38 % [15–17, 19]. The availability of more energy dense fast foods and exposure to sedentary life in Addis Ababa and its surroundings (compared to other urban settings in the country) might explain the lower figure for the country as a whole.

This finding is also lower than other studies based on DHS data; from Nigeria (26.7 and 36.4 %) [14, 27], and from seven African countries (average prevalence of 31 %) [6]. It is also lower than other pocket studies conducted in Benin (41.3 %), South Africa (56.6 %), Iran (61.3 %) and India (75.33 %) [28–31]. This might be due to disparity with the dietary pattern and life style, level of urbanization and economic development of Ethiopia.

In this study, age of women, marital status, wealth status, residence and educational status were found to be significantly associated with overweight and obesity. Consistent with the findings of other studies, the results of this study demonstrated that the prevalence was significantly higher among older women [16, 20, 27, 32, 33]. The decrease in level of physical activity and a higher intake of energy dense foods as age of women advances could be the possible explanation [34]. Similarly, the condition of being overweight and obese was significantly higher among women in the richest quintile which was consistent with studies from Addis Ababa and Wonji Shewa sugar factory [15, 16, 20] and studies from elsewhere [6, 27, 32, 33, 35]. In developing countries context the wealthier are likely to consume energy dense foods and follow a sedentary life style, hence they are more likely to be overweight and obese.

The prevalence of overweight and obesity was significantly higher among women at high school and above level, which is in line with other similar studies [6, 32, 34, 35]. This may be the result of shifts from manual labor to more sedentary occupations and the related decline in physical activity. Like in other studies overweight and obesity was more common among married women [6, 36]. Married are likely to have higher parity which could be linked to adopting more sedentary life style and high energy foods are usually offered for women during postpartum period.

The use of national and sub-nationally representative data and considerations of complex sampling methods are the strengths of this study. The following limitations should be considered while interpreting the results of this study. Firstly, cross-sectional nature of the study does not allow establishing causality of associations. Secondly, important predictors of the outcome variable such as physical activity and total energy intake (nutritional history) are not included owing to use of secondary data. Likewise, there was no data on central obesity since the survey did not collect data on abdominal and waist-to-hip circumference.

## Conclusions

The prevalence of overweight and obesity among urban women in Ethiopia was found to be considerably high compared to the national average. This was particularly more pronounced among older women, those in the richest quintile, with higher education, those living in the three metropolises (Addis Ababa, Dire Dawa and Harari) and married women. Based on the findings of this and other studies, it is imperative to design targeted overweight reduction programs with special emphasis on older, educated and well to do women, and those living in the big cities. There is also a need for more in-depth studies that account for important predictors including level of physical activity, nutritional history, and indicators of central obesity such as waist and abdominal circumference.

## Abbreviations

BMI, body mass index; CI, confidence interval; CSA, Central Statistical Agency; DALYs, global disability adjusted life years; DHS, Demographic Health Survey; EDHS, Ethiopia Demographic Health Survey; Kg/m ^2^, kilogram per meter square; SNNPR, Southern Nations Nationalities and Peoples Region; USAID, United States Agency for International Development
